# Neurophysiological and Neuroradiological Changes in Children With Chronic Kidney Disease

**DOI:** 10.3389/fped.2020.570708

**Published:** 2020-11-16

**Authors:** Sameh A. Abd El Naby, Wael A. Bahbah, Zeinab A. Kasemy, Asmaa A. Mahmoud

**Affiliations:** ^1^Department of Pediatrics, Faculty of Medicine, Menoufia University, Shebin El Kom, Egypt; ^2^Department of Public Health and Community Medicine, Faculty of Medicine, Menoufia University, Shebin El Kom, Egypt

**Keywords:** chronic kidney disease, uremic neuropathy, peripheral neuropathy, nerve conduction, electromyography, electroencephalography, magnetic resonance of brain

## Abstract

**Background:** Patients with chronic kidney disease (CKD) on maintenance hemodialysis frequently present with neurological complications. These complications include peripheral neuropathy, encephalopathy, and stroke.

**Objectives:** To detect the prevalence of neurological manifestations and complications in children with CKD through neurophysiological and neuro-radiological findings.

**Methods:** The study included 50 patients with CKD admitted to a pediatric nephrology unit. Their history and complete physical and neurological examination findings had been recorded. All patients underwent nerve conduction, electromyography, electroencephalography, and magnetic resonance imaging of the brain.

**Results:** Fifty children of both sexes (23 males and 27 females) with a mean age of (12.08 ± 3.46 year) were studied. Eleven (22%) patients with CKD developed polyneuropathy, mostly of an axonal polyneuropathy pattern, while 39 (78%) of them showed normal electrophysiological studies. No myopathy was detected. Abnormal electroencephalography findings were detected in 18% of patients, mostly generalized and focal (temporal, occipital, and frontal) epileptogenic activity. Abnormal MRI brain findings were detected in 16% of patients, mostly of encephalomalacia.

**Conclusion:** Uremic neuropathy was highly prevalent in children with CKD on maintenance hemodialysis. They developed polyneuropathy, mostly of an axonal polyneuropathy pattern. EEG is a useful method for early recognition of subclinical uremic encephalopathy and/or epileptogenic activity. Early demonstration and management of uremic neurological conditions may decrease the physical disability of CKD patients.

## Introduction

The National Kidney Foundation's Kidney Disease and Outcome Quality Initiative (KDOQI) Group defined chronic kidney disease (CKD) as when the glomerular filtration rate (GFR) is <60 mL/min per 1.73 m^2^ for ≥ 3 months or when the GFR is ≥ 60 mL/min per 1.73 m^2^ if other evidence of kidney damage exists, which is manifested by pathologic abnormalities reported by renal biopsy or abnormalities detected by urine, blood tests, and imaging procedures (1). CKD may be caused by a primary renal disorder or a multisystem disorder complication, such as diabetes. Neurological features and changes in children with CKD are highly prevalent whatever the cause (2). Encephalopathy detected in patients with chronic kidney disease results from their exposure to several factors, such as uremia, hypertension, and fluid, and electrolyte disturbances (3). Uremic encephalopathy features include alterations of mental status (alertness and awareness alterations, poor concentration, psychosis, and hallucinations, without treatment of which stupor, and coma may develop) and motor system abnormalities, such as clouding of the sensorium as an early feature and delirium, seizures, and coma as late features (4). Dialysis disequilibrium syndrome's neurological manifestations include headache, blurred vision, muscle cramps, tremors, disturbed consciousness, and convulsions, which are caused by the rapid removal of urea during hemodialysis. Rapid removal of urea from blood due to rapid hemodialysis resulting in osmotic gradients between the plasma and the brain that leads to cerebral edema which leads to these neurological manifestations (5). Peripheral neuropathy or uremic neuropathy affects 60–90% of CKD dialysis patients, so it is considered the most common neurological complication (6). Early clinical features of uremic neuropathy are numbness and paresthesia, resulting from large myelinated sensory fibers' affection. Distal sensory loss in the lower limbs and decreased or absent deep tendon reflexes are detected by clinical examination. Motor involvement showing weakness and muscle atrophy is associated with more severe conditions (7). When GFRs levels decrease below 12 ml/min, clinical manifestations of uremic neuropathy become clear (8). The uremic myopathy mechanism in CKD is unclear but may occur due to the role of oxidative stress (9). If the GFR decreases below 25 mL/min with an impairment of kidney function, uremic myopathy clinical features will appear (10) as proximal muscle weakness in the muscles of the lower limbs, leading to limited function of these muscles (11). Stroke displays a highly prevalent pattern in patients with CKD. Its prevalence is 17% for long term hemodialysis CKD patients, and 10% for mild to moderate CKD patients. Silent brain infarcts on magnetic resonance imaging (MRI) of the brain have been found in up to 50% of progressive stages of CKD (12). Hypertension, hypercholesterolemia, and disorders of mineral and bone metabolism increase the risk of stroke (13). So, we aimed to detect the prevalence of neurological manifestations and complications in children with chronic kidney disease through neurological examination and neurophysiological and neuro-radiological findings.

## Subjects and Methods

### Patients

A cross-sectional study with a cluster sample technique was carried out at a nephrology unit on 50 children aged <18 years old who presented with chronic kidney disease. Children included in this study had been on regular hemodialysis for more than 6 months. There were 23 males and 27 females with a mean age of (12.08 ± 3.46 year). Patients with central or peripheral nervous system disease from congenital or other causes other than CKD, and who had previous polyneuropathy and myopathy caused by thyroid dysfunction or diabetes mellitus, were excluded.

### Methods

All patients were subjected to history taking and complete physical and neurological clinical examination.

- Polyneuropathy was assessed through a number of factors. Clinical factors included numbness, tingling, insomnia, pain, or paresthesia. Patients were also examined for the detection of distal motor wasting, weakness, hypotonia, and hyporeflexia. Nerve conduction and electromyography (EMG) were also used to confirm the diagnosis and detect the type of polyneuropathy (axonal or demyelinating). Myopathy was assessed clinically through the detection of proximal weakness and wasting, and electro-physiologically by nerve conduction and (EMG) studies that confirm the diagnosis and determine the muscle affected. All studied patients underwent electroencephalography (EEG) and magnetic resonance imaging of the brain. A nerve conduction study was carried out on 50 patients with CKD and 50 healthy children matched for age, gender, residence, and socioeconomic state as a control group.
- Serum creatinine, blood urea nitrogen, and albumin were measured for all subjected patients. Calculation of GFR according to the modified Schwartz formula was done for all patients: GFR (mL/min/1.73 m^2^) = height (cm) × 0.413/serum creatinine (mg/dL). Normal value of eGFR was ≥ 90 ml/min/1.73 m^2^ and decreased value of eGFR was <90 ml/min/1.73 m^2^ (14).

### Neurophysiological Studies

- **Nerve conduction and electromyography (EMG):** All patients in the current study underwent a motor nerve conduction study of both tibial and peroneal nerves on both sides and a sensory nerve conduction study of both sural nerves. Eleven patients diagnosed as having polyneuropathy underwent a motor and sensory nerve conduction study of both median and ulnar nerves on both sides, which showed normal nerve conduction study. An EMG study of the gluteus maximus, tibialis anterior, and gastrocnemius (medial head) muscles on both sides had been performed.
- **Diagnostic criteria of polyneuropathy and myopathy:** Myopathy is detected through normal nerve conduction studies, and is characterized by the presence of small, polyphasic motor units with short durations and early recruitment patterns in both gluteus maximus muscles (15). Axonal polyneuropathy is characterized by decreased compound muscle action potential and sensory nerve action potential to <80% of normal in two or more nerves with no conduction block and normal conduction velocities with an EMG study that illustrates denervation potential, large polyphasic motor units, and decreased interference patterns in both the tibialis anterior and gastrocnemius muscle. Acute demyelination polyneuropathy is characterized by a prolonged distal motor latency to more than 115% of the upper limit of normal and low conduction velocity to <90 % of the lower level of normal with the presence of conduction block (16). Diagnosis of acute demyelination polyneuropathy by EMG study demonstrates no denervation potential at rest in the distal muscle, normal motor unit, and decreased recruitment pattern.
- **Electroencephalogram:** An electroencephalogram was carried out with a recording time of 20 min under standard conditions using Galileo Sirius 9231134, from 19 scalp electrodes, with average reference, according to the International 10–20 System (17).

### Statistical Analysis

Data were analyzed using IBM SPSS statistics version 22.0 (SPSS Inc., Chicago, IL, USA). Percentage (%), mean, and standard deviation (SD) were used for the descriptive statistics. For quantitative data, comparison between two means was done using Student *t-*test. For comparison of not normally distributed variables, Mann-Whitney was used. Fisher's exact test was used for qualitative data when the expected value is < 5. *P* < 0.05 is considered significant. Receiver operating characteristic (ROC curve) is a graphical plot of the sensitivity, vs. false positive rate (one minus the specificity). Sensitivity or true positive rate (TPR) = True +ve/(True +ve + False –ve). Specificity (SPC) or True Negative Rate = True –ve/(True –ve + False +ve) = 1- False +ve rate. Accuracy (ACC) = (True +ve) + (True –ve)/(Positive + Negative). Positive predictive value (PPV): True +ve/(True +ve + False +ve). Negative predictive value (NPV): True –ve/(True –ve + False –ve). Positive Likelihood Ratio (+LR) = sensitivity/(100–specificity). Negative Likelihood Ratio (–LR) = (100–sensitivity)/specificity.

## Results

Demographic features and clinical and neurological examinations are illustrated in (Table 1). Seizures were reported in 22% (*n* = 11), headache in 24% (*n* = 12), numbness in 18%(*n* = 9), dizziness in 10 % (*n* = 5), insomnia in 14% (*n* = 7), memory disturbance in 6% (*n* = 3), delayed speech in 8 % (*n* = 4), hypotonia in 22% (*n* = 11), hyporeflexia in 22% (*n* = 11), and hyperreflexia in 10% (*n* = 5) of children with CKD. The prevalence of peripheral neuropathy among the studied patients detected through electrophysiological study was 22% (*n* = 11 of 50), axonal motor and sensory neuropathy was observed in 81.8% (*n* = 9), and demyelinating motor neuropathy was reported in 18.2% (*n* = 2). By the analysis of the nerve conduction study, the results illustrated a high significant difference between both groups regarding amplitude. There was marked reduction of the amplitude of tibial, peroneal, and sural nerves in the patient group. Also, there was marked reduction in conduction velocity in the patient group in comparison to the control group of tibial, peroneal nerves, and right sural nerve. There was a high significant difference concerning the distal motor latencies in both groups (Table 2).

**Table 1:** Distribution of demographic, clinical, and neurological examinations in patients with CKD.

**Studied variables**	**Patients (*N =* 50)**	
**Age/years**		
**Mean ± SD**	12.08 ± 3.46	
**Range**	4–18	
**Studied variables**	**no**	**%**
**Sex**		
- Male	23	46.0
- Female	27	54.0
Seizures	11	22.0
Headache	12	24.0
Numbness	9	18.0
Dizziness	5	10.0
Insomnia	7	14.0
Disturbance of memory	3	6.0
Normal mentality	50	100.0
Delayed speech	4	8.0
Absent cranial nerve palsy	50	100.0
Hypotonia	11	22.0
Decreased power	15	30.0
**Reflexes**		
- Hyperreflexia	5	10
- Hyporeflexia	11	22
- Normal reflexes	34	68
**Peripheral neuropathy**		
Negative	39	78.0
Positive	11	22.0
**Positive**	*N =* 11	
- Axonal	9	81.8
- Demyelinating	2	18.2

**Table 2:** Nerve conduction findings among patients and controls.

**Nerve conduction**	**Patients *N =* 50**	**Controls *N =* 50**	***t-*test**	***P-*value**
	**Mean ± SD**	**Mean ± SD**		
**Rt tibial nerve**				
CV (m/s)	44.06 ± 6.64	47.6 ± 4.68	3.12	0.002^*^
Latency (ms)	5.10 ± 1.07	3.90 ± 0.81	6.29	<0.001^*^
Amplitude (mv)	12.00 ± 3.64	15.42 ± 1.51	6.14	<0.001^*^
**Lt tibial nerve**				
CV (m/s)	39.72 ± 4.36	52.76 ± 4.51	14.71	<0.001^*^
Latency (ms)	5.46 ± 0.86	3.94 ± 0.84	8.91	<0.001^*^
Amplitude (mv)	11.32 ± 3.42	17.04 ± 1.97	10.24	<0.001^*^
**Rt peroneal nerve**				
CV (m/s)	40.80 ± 4.40	53.24 ± 4.63	13.77	<0.001^*^
Latency (ms)	5.28 ± 0.97	4.34 ± 0.98	4.82	<0.001^*^
Amplitude (mv)	3.64 ± 1.30	4.84 ± 0.86	4.65^#^	<0.001^*^
**Lt peroneal nerve**				
CV (m/s)	40.04 ± 4.72	53.30 ± 4.52	14.34	<0.001^*^
Latency (ms)	5.42 ± 1.00	4.00 ± 0.81	7.80	<0.001^*^
Amplitude (mv)	3.77 ± 1.22	4.94 ± 0.82	5.61	<0.001^*^
**Rt sural nerve**				
CV (m/s)	50.90 ± 9.66	60.20 ± 7.95	5.26	<0.001^*^
Latency (ms)	3.50 ± 0.73	2.86 ± 0.73	4.37	<0.001^*^
Amplitude (mv)	5.42 ± 1.68	6.50 ± 0.81	4.09	<0.001^*^
**Lt sural nerve**				
CV (m/s)	51.40 ± 8.15	51.60 ± 8.89	0.12	**0.91**
Latency (ms)	2.90 ± 0.73	2.90 ± 0.73	0.00	**1.00**
Amplitude (mv)	5.54 ± 1.66	6.44 ± 0.73	3.51	0.001^*^

# Mann-Whitney test; CV, Conduction velocity; Rt, right; Lt, left;

* *significant*.

*Bold values indicates significant p-values*.

When analyzing the EMG study, the results revealed normal motor units in 41 patients (82%), while nine patients (18%) revealed large polyphasic motor units (neuropathic) as regards the tibialis anterior muscle and medial head of the gastrocnemius muscle, while all studied patients (100%) revealed normal motor units as regards the gluteus maximus muscle. There was a decreased interference pattern in 11 patients (22%) and denervation potential in the tibialis anterior and medial head of the gastrocnemius muscles in nine patients (18%). Abnormal EEG findings were demonstrated in 18% (*n* = 9) of studied subjects; 44.4% (*n* = 4) had generalized epileptogenic activity and 55.6% (*n* = 5) had focal epileptogenic activity (40% temporal, 40% occipital, and 20% frontal). EEG background was normal in 80% (*n* = 40) and diffuse slowing (of high voltage theta and delta waves) in 20% (*n* = 10) of studied patients with elevated serum creatinine. 60% (*n* = 6) of studied patients who had diffuse slowing presented with peripheral polyneuropathy. Abnormal brain MRI findings were reported in eight patients (16%), including mild brain atrophy in 37.5% (*n* = 3) and encephalomalacia in 62.5% (*n* = 5) (Table 3).

**Table 3:** Description of Electromyography, EEG, and Brain MRI findings among patients.

	**Patients (*N =* 50)**	
	**No**.	**%**
**Electromyography findings**		
**Tibialis anterior muscle**		
**Motor unit**		
Normal	41	82
Large polyphasic	9	18
**Interference**		
Normal	39	78
Decreased	11	22
**Denervation**		
No	41	82
Yes	9	18
**Gastrocnemius muscle medial head**		
**Motor unit**		
Normal	41	82
Large polyphasic	9	18
**Interference**		
Normal	39	78
Decreased	11	22
**Denervation**		
No	41	82
Yes	9	18
**EEG background**		
Normal	40	80
Diffuse slowing	10	20
**Diffuse slowing findings**	(*N =* 10)	
Peripheral Neuropathy	6	60
Normal	4	40
**EEG findings**		
Normal	41	82
Abnormal	9	18
**Abnormal EEG**	(*N =* 9)	
Generalized epileptogenic activity	4	44.4
Focal epileptogenic activity	5	55.6
**Focal epileptogenic activity**	(*N =* 5)	
Temporal	2	40
Frontal	1	20
Occipital	2	40
**Brain MRI**		
Normal	42	84
Abnormal	8	16
**Abnormal brain MRI**	(*N =* 8)	
**Mild brain atrophy**	3	37.5
**Encephalomalacia**	5	62.5

*Gluteus Maximus muscle had normal motor unit interference and innervation in all patients*.

Numbness, decreased eGFR, and increased serum creatinine had significant relation to peripheral neuropathy in patients with CKD (Table 4).

**Table 4:** Distribution of demographic, clinical, and neurological examination in patients with CKD with and without peripheral neuropathy.

	**Polyneuropathy**				**Test of sig**	***P-*value**
	**Yes** ***N =*** **11**		**No** ***N =*** **39**			
	**Mean ± SD**		**Mean ± SD**			
**Age (Years)**	11.36 ± 3.35		12.28 ± 3.50		0.75	0.450
**Sex**						
Male	4	36.4	19	48.7	0.52	**0.468**
Female	7	63.6	20	51.3		
**Seizure**	2	18.2	9	23.1	0.12	**1.0**
**Headache**	3	27.3	9	23.1	0.08	**1.0**
**Numbness**	9	81.8	0	0.0	38.91	<0.001^*^
**Dizziness**	0	0.0	5	12.8	1.56	0.573
**Insomnia**	2	18.2	5	12.8	0.20	**0.641**
**EEG abnormality**	3	27.3	6	15.4	0.82	**0.392**
**MRI abnormality**	3	27.3	5	12.8	1.33	**0.351**
**Albumin (g/dL)**	4.88 ± 0.25		4.08 ± 0.27			
Median	4.90		4.0		4.83	<0.001^*^
IQR	4.60–5.10		3.80–4.30			
**eGFR (ml/min/1.73 m**^**2**^**)**	16.63 ± 3.23		25.74 ± 1.66			
Median	16.0		26.0		5.07	<0.001^*^
IQR	14–20		25–27			
**Creatinine (mg/dl)**	8.31 ± 0.64		6.25 ± 0.81			
Median	8.20		6.30		5.01	<0.001^*^
IQR	7.80–8.90		5.70–7.0			

ann-Whitney test; CV, Conduction velocity; Rt, right; Lt, left;

* significant.

*Bold values indicates significant p-values*.

**Figure 1:**
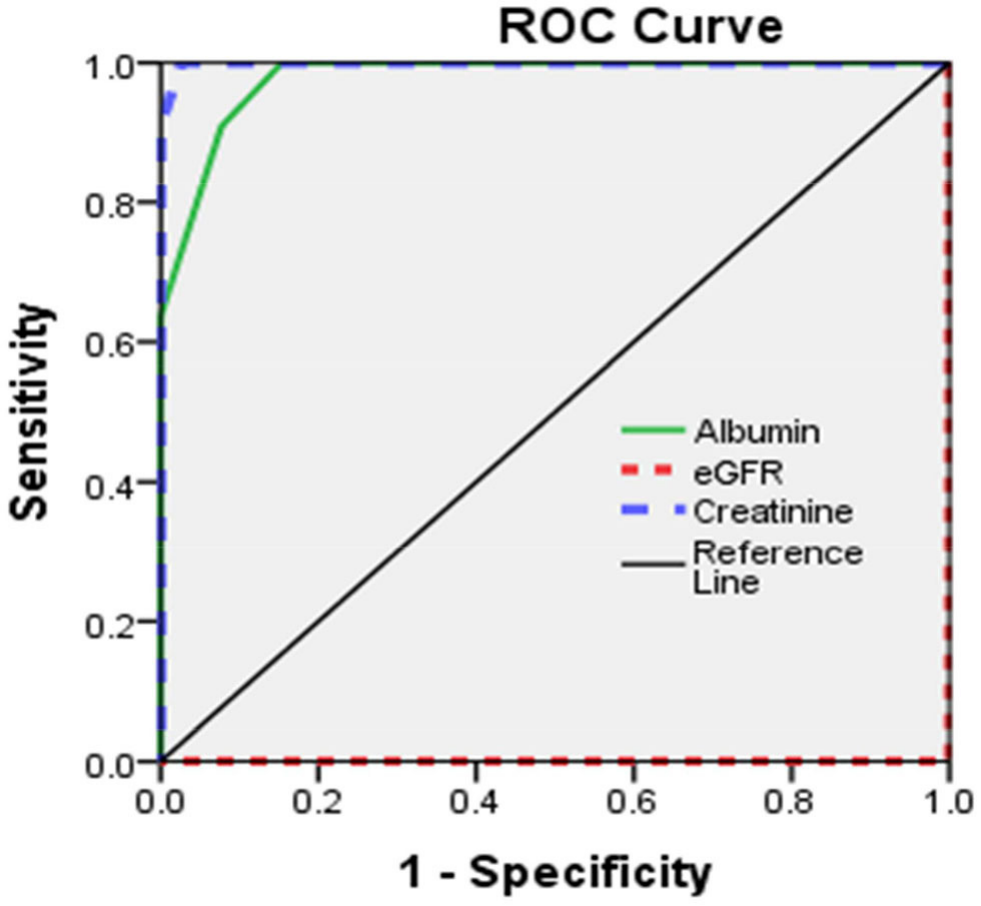
Roc curve for eGFR, Albumin, and serum creatinine in relation to Peripheral neuropathy.

Sensitivity and specificity of eGFR, serum creatinine, and serum albumin were illustrated (Table 5, Figure 1).

**Table 5:** Validity of albumin, eGFR, and creatinine in diagnosis of peripheral neuropathy.

	**Albumin** **g/dL**	**eGFR** **ml/min/1.73 m^**2**^**	**Creatinine** **mg/dl**
Area under curve	0.97	1.0	0.99
Cutoff point	>4.40	<21.5	>7.45
Sensitivity	100%	100%	100%
Specificity	85%	100%	97%
Accuracy	88%	100%	98%1
PPV	65%	100%	91.7%
NPV	100%	100%	100%
+LR	6.67	–	33.33
–LR	0	–	–

## Discussion

Sensorimotor axonal polyneuropathy caused by axonal degradation and loss of myelin sheath is common and affects the lower limbs (18, 19). The accumulation of uremic toxins attributed to oxidative stress leads to motor, sensory, and autonomic nerve damage, resulting in uremic neuropathy (20, 21). Chronic uremic nerve depolarization contributes to uremic neuropathy (UN) and is caused by hyperphosphatemia and hyperkalemia. Axonal death results from potassium disruption of the normal ionic gradient (22).

Seizures were reported in 11 patients (22%) due to epilepsy and uncontrolled hypertension and headache in 12 patients (24%) due to uncontrolled hypertension; both were the major central neurological manifestation in patients with CKD. This is in accordance with both Scorza et al. (23) who reported five cases with seizures and Afsharkhas et al. (24) who reported that 12 (40%) patients had neurologic findings including seizures in seven (23.4%) patients due to epileptic syndromes, hypertension with posterior reversible encephalopathy syndrome, febrile convulsion, and increased intracranial tension.

In the current study, neurological presentation of uremic neuropathy varies from numbness, insomnia, weakness, hypotonia, and hyporeflexia. Early symptoms of UN are paresthesia, increased pain sensation, and cramps. Long-term symptoms of UN are weakness, numbness, impaired deep tendon reflexes, and lower limb atrophy (25, 26).

The prevalence of peripheral neuropathy [uremic neuropathy (UN)] in the current study was 22%. Axonal motor and sensory neuropathy represented 81.8%, while demyelinating motor neuropathy represented 18.2%. Some studies reported UN prevalence ranging from 0 to 59% (27). Yoganathan et al. (28) demonstrated that uremic neuropathy prevalence was 52%, with axonal neuropathy (80.8%) and demyelinating neuropathy (11.5%) with predominant motor neuropathy. Ackil et al. (20) showed that 59% of children had sural nerve conduction abnormalities, and motor conduction abnormality was found in 29% of children on maintenance dialysis. The most common affected nerves were the tibial and common peroneal nerves as there was reduced conduction velocity and amplitude in our patients; this is in agreement with Yoganathan et al. (28). Another study reported that low peroneal nerve conduction velocity was considered as a useful tool for uremic neuropathy detection (29). De Camargo et al. (30) demonstrated that children with mild renal failure presented with a significant decrease of peroneal motor nerve conduction velocity.

The current study illustrated normal motor and sensory nerve conduction of both the ulnar and median nerves on both sides in our polyneuropathy patients; this is in agreement with Yoganathan et al. (28). Analytical interpretation of EMG results of the current study revealed normal motor units in 41 patients (82%), while nine patients (18%) revealed large polyphasic motor units (neuropathic). All studied patients (100%) revealed normal motor units as regards the gluteus maximus muscle. There was a decreased interference pattern in 11 patients (22%) and denervation potential in the tibialis anterior and medial head of the gastrocnemius muscles in nine patients (18%). Fahal and Bell reported normal electromyography (EMG) findings in chronic kidney disease patients (11). Berretta et al. (31) illustrated lower extremities proximal muscle weakness, particularly pelvic girdle proximal musculature, and EMG findings illustrated high-voltage polyphasic potentials in a boy with poor kidney function with elevated BUN (blood urea nitrogen) and creatinine. Abnormal EEG findings were demonstrated in 18% (*n* = 9) of the studied subjects, which were distributed as 44.4% (*n* = 4) with generalized epileptogenic activity and 55.6% (*n* = 5) with focal epileptogenic activity (40% temporal, 40% occipital, and 20% frontal). EEG background was normal in 80% (*n* = 40) and diffuse slowing (of high voltage theta and delta waves) in 20% (*n* = 10) of studied patients with elevated serum creatinine. Six patients (60%) of those who had diffuse slowing (*n* = 10) were presented by peripheral polyneuropathy. Gadewar et al. (32) showed that, with progression of CKD stage, frontal sharp wave transients were reported in 71.43% of patients at stage 4 and in 50% of those at stage 5. They found a high theta pattern in both stages. Demir et al. (33) reported that bilateral spike wave activity was found in 14% of patients and decreased alpha activity in 8.5% of patients with uremic encephalopathy. Also, Koçer et al. (34) demonstrated that occipital sharp wave transients were found in stage 4 of CKD. While Demir et al. (33) and Burn and Bates (35) demonstrated generalized slowing and bilateral spike and wave complexes in 14% of patients, Arnold and Krishnan reported that the triphasic sharp waves were the typical features of EEG abnormalities found in uremic encephalopathy (36). EEG typical presentations in uremic encephalopathy are a slow alpha rhythm with excess delta and theta waves (10). Lai et al. (37) illustrated significant differences in EEG findings of the relative power of the theta band and absolute and relative power of the delta band. Arieff (38) showed the appearance of theta waves, disappearance of normal basic rhythms, and diminished reactivity of EEG to afferent stimulation and domination by generalized delta activity. Röhl et al. (39) showed prominent electrical power in theta, delta, and alpha frequencies in the temporal and central brain areas. Abnormal MRI findings were reported in 16% of studied subjects (*n* = 8), including mild brain atrophy in 37.5% (*n* = 3) and encephalomalacia in 62.5% (*n* = 5). This may be due to hypertensive encephalopathy, uremia, electrolytes disturbance, seizure, or hypoxia. Ishikura et al. (40) reported radiological abnormalities extending to 85% of gray matter, especially in the frontal and temporal lobes. Brain atrophy was estimated in three (23%) of 13 children who presented with chronic renal failure (27). The current study reported a significant association of eGFR, serum creatinine, and albumin with peripheral polyneuropathy with sensitivity (100% for all) and specificity (100, 97, and 85%, respectively). Yoganathan et al. (28) reported the sensitivity was 84.6% (95% CI 66.47, 93.85) and the specificity was 69.6% (95% CI 49.13, 84.4) for age, serum albumin, eGFR, serum copper and ferritin, and their relation to peripheral neuropathy prediction.

### Limitations of the Study

Autonomic neuropathy was not evaluated as any clinical manifestations, whether digestive, visual, urinary, body temperature, heart, or blood vessel symptoms, were not included in the studied patients. We recommend tilt-table, quantitative sudomotor axon reflex, and urodynamic tests and also the cutaneous sympathetic response as a rapid screening tool for the involvement of the autonomic nervous system.

## Conclusion

Uremic neuropathy is highly prevalent in children with CKD on maintenance hemodialysis. They developed polyneuropathy was mostly of an axonal polyneuropathy pattern. EEG is a useful method for early recognition of subclinical uremic encephalopathy and/or epileptogenic activity. Early demonstration and management of uremic neurological conditions may decrease the physical disability CKD patients.

## Data Availability Statement

All datasets generated for this study are included in the article/Supplementary Material.

## Ethics Statement

The studies involving human participants were reviewed and approved by The Institutional Review Board (IRB) of the Menoufia Faculty of Medicine approved the study (ID: 190719Ped). Research work was performed in accordance with the Declaration of Helsinki. A written patient consent was taken from the parents and caregivers after explaining all aspects of the study, with the right to withdraw at any time. Written informed consent to participate in this study was provided by the participants' legal guardian/next of kin. Written informed consent was obtained from the individual(s), and minor(s)' legal guardian/next of kin, for the publication of any potentially identifiable images or data included in this article.

## Author Contributions

All authors co-operated in conceptualization, design of the work, data collection, resources detection, formal analysis, interpretation of data, the creation of new software used in the work, validation, methodology, and revision. SA, WB, and AM collected the data and wrote the manuscript. ZK analyzed the data, drafted, and revised the manuscript.

## Conflict of Interest

The authors declare that the research was conducted in the absence of any commercial or financial relationships that could be construed as a potential conflict of interest.

## Abbreviations

UN: Uremic neuropathy
EMG: Electromyography
EEG: Electroencephalography
MRI: Magnetic Resonance imaging.
